# Indications of airway stenting for severe central airway obstruction due to advanced cancer

**DOI:** 10.1371/journal.pone.0179795

**Published:** 2017-06-26

**Authors:** Hiroaki Nagano, Tomoo Kishaba, Yuichirou Nei, Shin Yamashiro, Hiroaki Takara

**Affiliations:** 1 Department of Respiratory Medicine, Okinawa Chubu Hospital, Okinawa, Japan; 2 Department of Radiology, Okinawa Chubu Hospital, Okinawa, Japan; Seoul National University College of Pharmacy, REPUBLIC OF KOREA

## Abstract

**Background:**

Management of severe central airway obstruction due to advanced cancer is a medical and technical challenge. The impact of airway stenting on the clinical outcome of such patients is unclear.

**Method:**

This single-center, retrospective study evaluated 21 patients who underwent airway stenting for advanced cancer. We examined predictors of the post-stenting mortality, including age, serum albumin, tracheal diameter, smoking, opioid use, respiratory failure, and performance status (PS). We also compared survival according to the PS.

**Results:**

The mean survival period after stenting was 85.2 days. On univariate analysis, age, albumin, PS before airway stenting, respiratory failure, admission route, and PS grade were the candidates as possible predictors of prognosis after the procedure. On multivariate analysis, PS before airway stenting was identified as possible predictor of prognosis after stenting (HR 1.6180, 95% CI 0.969 to 2.7015, p = 0.066). The mean survival period after stenting was significantly longer in the good PS group, compared to the poor PS group (147.8 days vs. 38.2 days,p = 0.0346).

**Conclusion:**

Airway stenting for advanced cancer may be more effective for patients in good general condition than in those with poor performance status.

## Introduction

For pulmonologists, helping patients who have severe central airway obstruction (CAO) due to unresectable advanced cancer is a challenge because the condition can deteriorate anytime and lead to choking and sudden death. Dyspnea due to airway stenosis is an intolerable symptom for these patients [1]. Airway stenting is one of the options to relieve airway obstruction. However, the patient characteristics associated with the best outcomes after airway stent insertion remain to be defined [2,3].

We sometimes have the consult about the indication of tracheal stent insertion. Since there are no global standard guidelines, we often face the situations which is difficult to decide whether stent insertion benefit patients.

In the previous report, there are some conditions for stent placement [4]. However, these criteria are empirical and their evidence level is low.

The aim of this study was to evaluate the factors associated with mortality after airway stenting and to assess the outcome and efficacy of airway stenting for malignant CAO.

## Materials and methods

### Data collection

We retrospectively reviewed the medical records of patients who underwent airway stent insertion for severe airway obstruction caused by malignant tumor compression or invasion at our institution from January 1, 1998 to March 31, 2015. We investigated the patients’ background such as gender, age, smoking rate, primary organ, treatment before and after stenting, laboratory data, opioid use, airway diameter and procedure time. We investigated airway stenting outcomes, such as survival after stenting, discharge rate, and improvement of oxygenation.

We also focused on the patient’s performance status (PS), tried to investigate whether it affected survival or symptom palliation. The study population was divided into good PS **(PS:0–2)** and poor PS **(PS: 3–4)** groups. The reason why we divided patients into two groups is most chemotherapy regimens are not recommended for PS 3 and PS 4 patients in NCCN (National Comprehensive Cancer Network) guideline [5]. When we considered something invasive treatment for cancer, we decided that grouping of PS 0–2 and 3–4 is generally reasonable. Grouped PS was defined to analyze how each PS group (PS 0–2 and PS 3–4) was related to the patients’ survival.

### Airway stenting

**Fig 1:**
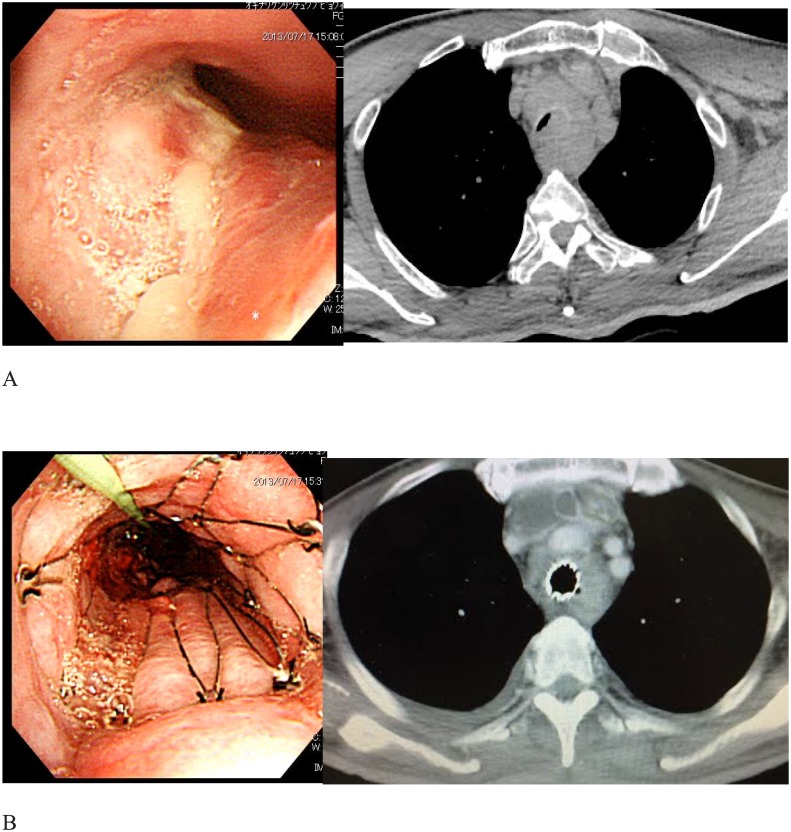
Airway stenting by metallic stent. (A) Tracheal compression from advanced esophageal cancer producing marked tracheal compression and critical airway stenosis. (B) Palliation by placement of tracheal Spiral Z stent producing immediate resolution of airway symptoms.

In all patients, metallic airway stents were used. Before procedure, benzodiazepines were used for sedation. Morphine was used for analgesia. After careful inspection of the tracheal lumen, the pulmonologists deployed the stent by flexible bronchoscopy. With the cooperation of the radiologists, the position of the airway stent was adjusted to the appropriate location under X-ray guidance “Fig 1”.

### Performance status, respiratory failure, oxygenation

The Eastern Cooperative Oncology Group Performance Status (PS) scores that were gathered from medical and nursing records were used to evaluate the activities of daily living of each patient [6]. Grade 0 was defined as fully active, able to carry on all pre-disease performance without restriction. Grade 1 was defined as restricted in physically strenuous activity, but ambulatory and able to carry out any work of light or sedentary nature. Grade 2 was defined as ambulatory and capable of all self-care, but unable to carry out any work activities; up and about for more than 50% of waking hours. Grade 3 was defined as capable of limited self-care only; confined to bed or chair for more than 50% of waking hours. Grade 4 was defined as completely disabled; cannot perform any self-care; totally confined to bed or chair. The study population was divided into good PS **(PS:0–2)** and poor PS **(PS: 3–4)** groups.

Respiratory failure was defined as severe dyspnea, respiratory distress, decreased oxygen saturation (less than 90% on room air), hypoxemia with PaO_2_<60mmHg, on room air, or requiem of noninvasive or invasive mechanical ventilation. The improvement of oxygenation was defined as the improvement of PaO_2_ on arterial blood gas, or decreased oxygen requirement.

### Mortality

Mortality was defined as survival two months after the stent insertion. This is because the mean survival period after stenting was 85.2 days.

### Ethics committee approval

The Ethics Committee of Okinawa Chubu Hospital approved the study protocol. Informed consent was waived because this study was a retrospective, epidemiologic, and anonymous review of medical records, rather than a prospective, interventional clinical study.

### Statistical analysis

Clinical data were presented as mean, depending on the distribution. Variables, such as clinical background, mean survival period, rate of discharge to home, post-procedure PS, improvement of oxygenation, and death from pneumonia, were compared between the good and poor PS groups using a X^2^ test. Univariate and Multivariate analysis with a Cox proportional hazards model was used to determine the relationships between the clinical parameters and survival after the procedure, as well as survival according to PS. We chose the objects for multivariate analysis which p-value were under 0.1 in univariate analysis. The level of statistical significance was set at P <0.05. STATA software V.11.0 (Stata Corp., College Station, TX, USA) was used for all statistical analyses.

## Results and discussion

### Clinical characteristics

This 15-year study included 21 patients who underwent airway stenting for malignant CAO. The study population had a mean age of 66.1 years and comprised 3 (14.3%) women and 17 (81.0%) patients who were smokers. The cause of CAO was esophagus cancer in 10 (50.0%), lung cancer in 7 (33.3%), metastatic lung cancer in 2(9.53%), malignant mesothelioma in 1 (4.76%) and hypopharyngeal cancer in 1(4.76%).

The classification of pathology was squamous cell carcinoma in 16 (76.2%), adenocarcinoma in 2 (9.5%), small cell carcinoma in 2 (9.5%), and malignant mesothelioma in 1 (4.8%). There were 10(47.6%)patients who had chemotherapy, 7(33.3%)patients who had radiation therapy before procedure. 2(9.5%) patients had both chemotherapy and radiation therapy before and after airway stent insertion. The rate of patients who had radiation therapy before procedure was significantly higher in good PS group than that of patients in poor PS group (66.7% vs 8.3%, p = 0.019). There were 7(33.3%) patients who underwent surgery before procedure, and there were no patients who had surgery after stent insertion.

There were 14 (66.7%) patients admitted to the hospital from the emergency department. The good PS group (n = 9) and poor PS group (n = 12) had similar background characteristics, such as gender, age, smoking rate, and laboratory data other than radiation therapy before procedure (Table 1).

**Table 1:** Background of patients who underwent airway stenting for malignant obstruction.

	All patients n = 21	Good PS(0–2) n = 9	Poor PS(3–4) n = 12	p value
Gender, n (%)	Male = 18(85.7%),Female = 3(14.3%)	Male = 8(88.9%),Female = 1(11.1%)	Male = 10(83.3%),Female = 2(16.7%)	0.735
Age, yrs, mean(range)	66.1(53–79)	64.2(56–72)	67.6(53–79)	0.3317
Smoking, n(%)	17(81.0)	8(88.9)	9(75)	0.4479
Primary lesion, n	Esophagus = 10,Lung = 7,Metastasis = 2,Mesothelioma = 1,Hypopharynx = 1	Esophagus = 5,Lung = 3,Hypopharynx = 1	Esophagus = 5,Lung = 4,Metastasis = 2,Mesothelioma = 1	
Pathology, n	Squamous = 16,Small = 2,Adeno = 2,Mesothelioma = 1	Squamous = 8,Small = 1	Squamous = 8,Adeno = 2,Small = 1 Mesothelioma = 1	
Chemotherapy before procedure, n (%)	10(47.6)	4(44.4)	6(50.0)	0.850
Chemotherapy after procedure, n (%)	5(23.8)	2(22.2)	3(25.0)	0.712
Radiation before procedure, n (%)	7(33.3)	6(66.7)	1(8.3)	0.019
Radiation after procedure, n (%)	5(23.8)	2(22.2)	3(25.0)	0.712
Surgery before procedure, n(%)	7(33.3)	3(33.3)	4(33.3)	0.640
WBC/μL mean(range)	9067(4300–12900)	8510(4300–12200)	9480(5500–12900)	0.4477
Alb g/dL,mean(range)	3.05(1.8–4.4)	3.0(1.8–4.4)	3.06(1.8–3.9)	0.9229
LDH IU/L,mean(range)	197(123–656)	176(123–316)	283(153–656)	0.1394
Opioid use, n(%)	12(47.6)	4(44.4)	8(66.7)	0.279
Time from diagnosis to procedure, months (range)	23.9(1–108)	23.3(1–108)	24.3(1–108)	0.9447
Airway diameter before procedure, mm(range)	4.3(1–8)	5.0(2–8)	3.9(1–8)	0.094
Procedure time, min, mean(range)	52(16–130)	59(16–130)	46.8(25–85)	0.2362

Abbreviations: WBC white blood cell, LDH lactate dehydrogenase

### Prognosis after the procedure

The mean survival period after stenting was 85.2 days. 13 (61.9%) experienced relief of dyspnea or improved oxygenation; and 11 (52.4%) patients were discharged to home after stenting. On univariate analysis,age, albumin, PS before airway stenting, respiratory failure, admission route, and group PS were the candidates as possible predictors of prognosis after the procedure. Other factors, such as age, gender, serum albumin, respiratory failure, admission route did not predict mortality after airway stenting. The treatments for cancer such as chemotherapy, radiation therapy and surgery also did not predict mortality on univariate analysis.(Table 2).

**Table 2:** Cox proportional hazards regression analysis of mortality after airway stenting: Univariate analysis.

	HR (95% CI)	p value
Age	2.100 (5.352 to 9.552)	0.018
WBC	0.0350 (0.0239 to 0.169)	0.722
Alb	14.040 (76.105 to 104.185)	0.006
LDH	0.113 (0.0549 to 0.328)	0.591
Gender	132.278 (15.059 to 279.614)	0.076
PS level before procedure	58.617 (13.270 to 103.964)	0.014
Opioid	53.482 (162.924 to 55.961)	0.319
Tracheal diameter	0.521 (27.929 to 26.887)	0.969
Respiratory failure	5.143 (5.061to 15.346)	0.055
Admission from ER	43.911 (139.182 to 51.360)	0.0467
Chemotherapy before procedure	0.520 (0.169 to 1.603)	0.255
Chemotherapy after procedure	0.740 (0.202 to 2.700)	0.649
Radiation before procedure	1.0329 (0.317 to 3.361)	0.957
Radiation after procedure	0.494 (0.109 to 2.232)	0.359
Surgery before procedure	1.343 (0.436 to 4.139)	0.608
Smoking	28.764 (113.543 to 171.073)	0.677
Grouped PS (Good or Poor)	109.361 (8.795 to 209.928)	0.035

ER: emergency room

On multivariate analysis,PS before airway stenting was identified as possible predictor of prognosis after the procedure (HR 1.6180, 95% CI 0.969 to 2.7015, p = 0.066). Grouped PS was also the candidate as possible predictor of prognosis because of high HR (HR 3.1555, 95% CI 0.8550 to 11.6459, p = 0.085) (Table 3).

**Table 3:** Cox proportional hazards regression analysis of mortality after airway stenting: Multivariate analysis.

	HR (95% CI)	p value
Age	1.0073 (0.9369 to 1.0831)	0.843
Alb	0.4740 (0.1706 to 1.3166)	0.152
Gender	0.435 (0.05741 to 3.4194)	0.4431
PS level before procedure	1.6180 (0.969 to 2.7015)	0.066
Respiratory failure	1.0254 (0.9337 to 1.1261)	0.600
Admission from ER	1.9897 (0.8207 to 4.8236	0.128
Grouped PS (Good or Poor)	3.1555 (0.8550 to 11.6459)	0.085

The mean survival period after the procedure was significantly longer in the good PS group, compared to the poor PS group (147.8 days vs. 38.2 days,p = 0.0346). The discharge rate after the procedure was higher in the good PS group than the poor PS group (77.8% vs. 33.3%; p = 0.0457). Improvement of oxygenation after stenting showed no statistically significance between good PS group and poor PS group (77.8% vs. 50.0%.p = 0.5181) (Table 4).

**Table 4:** Outcomes of airway stenting for malignant obstruction.

	All patients, n = 21	Good PS(0–2) n = 9	Poor PS (3–4) n = 12	p value
Mean survival, days(range)	85.2(10–516)	147.8(10–516)	38.4(15–85)	0.0346
PS after stenting, score (range)	2.3(0–4)	1.3(0–4)	3.0(0–4)	0.0033
Discharge to home after stenting(%)	11(52.4)	7(77.8)	4(33.3)	0.0457
Improvement of oxygenation	13(61.9)	7(77.8)	6(50)	0.5181

Of the 21 patients, 9 (42.9%) died of infectious pneumonia and 4 (19.0%) died due to cancer progression. Other causes of death were massive hemoptysis caused by trachea-artery fistula, pneumothorax, myocardial infarction, mucus plug, and other unknown factors.

#### Weaning from mechanical ventilation

Of all patients, 9 (42.9%) were intubated before airway stenting; 7 of 9 (77.8%) were successfully extubated after stenting. Five patients underwent elective intubation during airway stenting, 2 could not be extubated post-stenting and eventually died (Table 5).

**Table 5:** Weaning from mechanical ventilation after airway stenting for malignant obstruction.

	All patients, n = 21	Good PS(0–2) n = 9	Poor PS (3–4) n = 12	p value
Intubated patients, n(%)	9(42.9)	4(44.4)	5(41.7)	0.313
Extubation after stenting, n	7	3	4	0.998

## Discussion

For patients who have advanced cancer, quality of life is as important as survival [7,8]. Our duty and sincere intention as clinicians is to alleviate disabling dyspnea, obstructive pneumonia, and acute suffocation in patients with malignant airway obstruction. Depending on the type of malignancy, options for non-surgical palliation and local control of tumor spread include chemotherapy and/or radiotherapy. However, despite chemotherapy and radiotherapy, patients may still develop life-threatening CAO, which might be an appropriate indication for interventional bronchoscopy [1]. Airway stent insertion may provide immediate and gratifying palliation that can rescue patients from imminent death and assure improvement in quality of life [7,9,10,11]. However, the patient characteristics associated with the best outcomes after airway stent insertion are unknown [2,3]. Being an invasive procedure, airway stenting indications have to be considered carefully. In a previous study, Matsuo et al. reported their indications for stent placement, as follows: 1) severe CAO presenting with dyspnea and flow limitation on a flow-volume curve; 2) prognosis will be prolonged by stent placement; and 3) peripheral airways and lungs are otherwise intact [4]. In this present study, only pre-stenting PS was identified by multivariate logistic regression analysis as a potential independent predictor of prognosis after stent insertion. Since we had small number of patients, the p values did not reach statistical significance on multivariate analysis. However, HR of group PS showed high value. Therefore, we thought both PS and before airway stenting and grouped PS tended to show the possible predictor of prognosis after stenting.

Similar to chemotherapy, airway stenting indications might be dictated by a patient’s PS [12]. Based on our results, patients who have good PS may benefit from stenting in terms of longer survival and improved oxygenation. Indeed, stent placement was shown to prevent acute suffocation, alleviate fear of sudden death, and provide patients sufficient time to undergo additional therapies, such as chemotherapy, radiotherapy, and palliative care [11].

Care must be taken in choosing the indications of airway stenting in patients with poor PS. Although the severe symptoms of dyspnea can be alleviated by stent insertion, survival after the procedure might not be prolonged. Before the procedure, the limitations and temporary effects of stenting must be disclosed. The choice of airway stent is usually based on operator preference. Removable stents are preferred because long-term complications, such as infection, obstructive granuloma, fistula, and migration, are common [13–18]. For this study population, we chose metallic stents because of the radiologists’ experience on metallic stents, as opposed to rigid bronchoscopy with silicone stents [18][19].

In this study, the median survival time after the procedure was quite different from that of previous reports [9,11,13,17,19,20]. This might be due to differences in patients’ general condition and clinical background between our study and the previous reports.

In both groups, there were no major complications during the procedure. However, after the procedure, 9 patients died of pneumonia, which might have been caused by mucus plugging of the stent. One patient died due to massive hemoptysis from a trachea-artery fistula. Although this also may be associated with stent placement, the extent of stent involvement in the bleeding is unknown. [21]

In our study, 75% of the intubated patients were successfully extubated. This result was of great benefit to the patients because they were able to return to the general ward or to be discharged home. In the 2 patients who were intentionally intubated during airway stenting and subsequently died without being extubated, the cause could be an indirect complication of the procedure. One potential complication is sudden suffocation during stent insertion, especially in patients who have extremely severe airway stenosis or edema. For such cases, preparations for extracorporeal membrane oxygenation (ECMO) can be made [22–24]. In this study, 2 patients were placed on bypass during airway stent insertion. At present, evidence on the value of these adjuncts during stenting is limited. More discussion and studies that verify the safety, appropriate analgesia, and indications for intubation and ECMO during the airway stenting are needed.

We recognize some limitations of our study. This was a single-center, retrospective, observational study. Without comparison to a placebo group, we were unable to conclude whether airway stenting itself contributed to survival benefit.

From January 1, 1998 to March 31, 2015 is a very long period. Only 21 patients were analyzed during this long period. Much progress has been made for cancer therapy during this period. However, in our study, the treatments for cancer such as chemotherapy, radiation therapy and surgery did not influence on mortality with univariate analysis.

The efficacy of airway stenting depends on appropriate patient selection, anatomic location of the obstruction, and the underlying pathology. Therefore, careful consideration should be given when evaluating the risks and benefits of stenting for each patient [14]. We also have to carry out discussions with the patient and family members, as well as the paramedical staff, regarding fulfillment of each patient’s end of life goals.

## Conclusions

The indications for airway stenting in patients with advanced cancer should take into account the patient’s PS, which was the only possible predictor of prognosis after stenting as shown by our study. Airway stenting for advanced cancer may be more effective in patients in good general condition than in patients with poor PS. Attending physicians have to communicate with each patient about his/her life expectancy and evaluate the benefits of airway stenting based on PS.
